# Supplementary material for: Splice-Junction-Based Mapping of Alternative Isoforms in the Human Proteome
Source: Cell Rep. Author manuscript; Available in PMC 2020 Jan 15. (PMC6961840; doi:10.1016/j.celrep.2019.11.026)
Supplement: 2 [file NIHMS1546469-supplement-2.zip › DF1/PXD009737/TestisTrypsin/TestisTrypsin_2_CLTCL1_LQEHFQLQNLGINPANIGFSTLTMESDKFICIR.pdf]

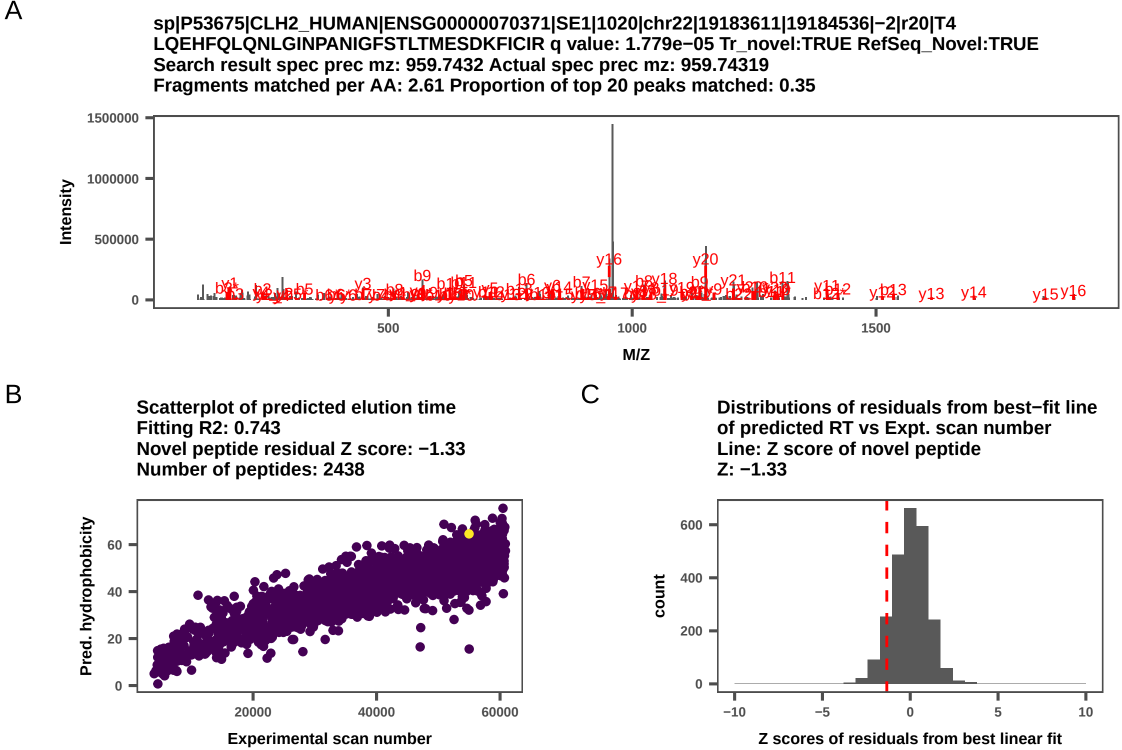
A
sp|P53675|CLH2_HUMAN|ENSG00000070371|SE1|1020|chr22|19183611|19184536|−2|r20|T4
LQEHFQLQNLGINPANIGFSTLTMESDKFICIR q value: 1.779e−05 Tr_novel:TRUE RefSeq_Novel:TRUE
Search result spec prec mz: 959.7432 Actual spec prec mz: 959.74319
Fragments matched per AA: 2.61 Proportion of top 20 peaks matched: 0.35
Intensity
1500000
1000000
500000
0
500
1000
1500
M/Z
y16
y20
b9
b6
b11
y13
y14
y15
y16
B
Scatterplot of predicted elution time
Fitting R2: 0.743
Novel peptide residual Z score: −1.33
Number of peptides: 2438
Pred. hydrophobicity
60
40
20
0
20000
40000
60000
Experimental scan number
C
Distributions of residuals from best−fit line
of predicted RT vs Expt. scan number
Line: Z score of novel peptide
Z: −1.33
count
600
400
200
0
−10
−5
0
5
10
Z scores of residuals from best linear fit
